# CREATING AN INTERGENERATIONAL COMMUNITY OF CAREGIVERS

**DOI:** 10.1093/geroni/igae098.1573

**Published:** 2024-12-31

**Authors:** Pamela Elfenbein

**Affiliations:** University of North Georgia, Oakwood, Georgia, United States

## Abstract

While the preponderance of those caregiving for older adults are over the age of 45, the 2020 Caregiving in the U.S. study, conducted by AARP and The National Alliance for Caregiving (NAC), revealed that 11 percent of Americans who are caring for older adults are also attending school either full-time or part-time. Adult Children of Aging Parents (ACAP) Hall County invites students, staff, faculty and community caregivers of all ages to meet monthly for information, resources, and support, building a community to enhance the quality of life for area caregivers and their older adult loved ones. Details of the program will be shared to enable presentation participants tp consider the development of a similar program on other campuses. This presentation aligns with AFU Principle 4:To promote intergenerational learning to facilitate the reciprocal sharing of expertise between learners of all ages.

